# Effects of pH and NaCl on the Spatial Structure and Conformation of Myofibrillar Proteins and the Emulsion Gel System—Insights from Computational Molecular Dynamics on Myosin of Golden Pompano

**DOI:** 10.3390/gels9040270

**Published:** 2023-03-24

**Authors:** Changfeng Xue, Zhisheng Pei, Pan Wen, Yaoxian Chin, Yaqin Hu

**Affiliations:** 1 Hainan Provincial Academician Team Innovation Center, Hainan Tropical Ocean University, Sanya 572022, China; 2 Marine Food Engineering Technology Research Center of Hainan Province, Hainan Tropical Ocean University, Sanya 572022, China; 3 Collaborative Innovation Center of Marine Food Deep Processing, Hainan Tropical Ocean University, Sanya 572022, China; 4 Yazhou Bay Innovation Institute, Hainan Tropical Ocean University, Sanya 572022, China

**Keywords:** myosin, myofibrillar protein, pH value, NaCl concentration, molecular dynamics simulation, emulsion gel

## Abstract

In this study, the effects of pH and NaCl concentrations on the structure of golden pompano myosin and emulsion gel were analyzed using SEM in combination with molecular dynamics simulations (MDS). The microscopic morphology and spatial structure of myosin were investigated at different pH (3.0, 7.0, and 11.0) and NaCl concentrations (0.0, 0.2, 0.6, and 1.0 M), and their effects on the stability of emulsion gels were discussed. Our results show that pH had a greater effect on the microscopic morphology of myosin than NaCl. The MDS results show that under the condition of pH 7.0 and 0.6 M NaCl, the myosin expanded and experienced significant fluctuations in its amino acid residues. However, NaCl showed a greater effect on the number of hydrogen bonds than pH. Although changes in pH and NaCl concentrations only slightly altered the secondary structures in myosin, they, nevertheless, significantly influenced the protein spatial conformation. The stability of the emulsion gel was affected by pH changes but not NaCl concentrations, which only affect the rheology. The best elastic modulus G″ of the emulsion gel was obtained at pH 7.0 and 0.6 M NaCl. Based on the results, we conclude that pH changes have a greater influence than NaCl concentrations on the spatial structure and conformation of myosin, contributing to the instability of its emulsion gel state. The data from this study would serve as a valuable reference for emulsion gel rheology modification in future research.

## 1. Introduction

Golden pompano (*Trachinotus ovatus*) is a teleost fish belonging to the order of Perciformes that serves as an important commercial fish in marine aquaculture. In China, the production of golden pompano reached 200,000 tons in 2020 and is valued at over CNY 5 billion [1]. Currently, golden pompanos are mainly sold whole as fresh, chilled, or frozen. The lack of product diversification underutilizes the fish, which is rich in polyunsaturated fatty acids and has a balanced ratio of amino acids [2]. Therefore, the deep processing and product diversification of golden pompano are of key concern to industrial players. As muscle tissue is the main factor affecting the taste and quality of fish meat, understanding the changes in muscle tissues when exposed to various treatments during food processing would ensure product quality. The salt-soluble myofibrillar protein, as the contractile component of muscle tissue [3], is responsible for many of its important properties, including its emulsification and gelation abilities. The solubility of myofibrillar protein is not only related to the protein structure but also to pH, ionic strength, temperature, and protein concentration as well. Due to the amphipathic nature of myofibrillar proteins, the hydrogen bonds and electrostatic and hydrophobic forces within the protein change in response to its surrounding environment, adopting a new equilibrium structure that leads to changes in its functional properties [4].

Myofibrillar proteins not only affect the sensory and texture quality of meat but also determine its emulsifying ability and water-holding capacity [5]. The emulsifying property of myofibrillar protein, as one of its most important characteristics, has been attracting significant research interest. Wu [6] prepared a Pickering-type high internal phase emulsion (O/W) using cod myofibrils that is strong (up to 1.6 MPa) and tough (up to 0.7 MJ m^−3^) and is suitable for food 3D printing. Li et al. [7] used pork myofibrillar protein to prepare a high internal phase emulsion (O/W) that is adaptable to various pH values, which could potentially act as a nutrient delivery system. In golden pompano, efforts have been made to understand and enhance the emulsifying capacity of its myofibrillar protein, including the effect of oxidation, pH changes, heat, and additives [2,8,9,10]. Various researchers have found that emulsion stability is strongly correlated to the interfacial rheological properties of protein membranes [11,12], and these properties are, in turn, affected by both the physicochemical characteristics of the adsorbed proteins (molecular flexibility, surface hydrophobicity, protein molecular size, etc.) and the environmental conditions (pH, ionic strength, protein concentration, etc.) [13]. Amphiphilic myosin, being the main protein component of myofibril, acts as the predominant emulsifier in pre-rigor meat due to its superior emulsifying capacity [14,15] and is generally believed to play a major role in the membrane–protein interface formation. Therefore, the properties of myosin molecules are of major interest when studying emulsion stability [16]. However, while myosin has been studied in some marine species [16,17,18], no study has been reported on its spatial conformation changes in golden pompano as far as the authors are aware. 

As emulsions have multiple interfaces, the actual state of proteins at the interfaces cannot be easily elucidated experimentally. Molecular dynamics simulation (MDS) is a powerful computer simulation method that allows the study of the physical motion of atoms and molecules, obtaining molecular and structural information at the atomic level. It mainly relies on Newtonian mechanics to simulate the motion of molecular components, calculating interaction forces within samples composed of different states of molecular systems and subsequently, the thermodynamics and other macroscopic properties of the system, including the configurations of proteins and other molecules [19]. The technology has become an important means to study the interaction mechanism of components, exploring the characterization and analysis of molecular properties that cannot be realized experimentally. It can be used to analyze and reasonably predict the conformation of proteins and their rearrangements in response to the surrounding environment and provide complementary information and allow better interpretation of the changes in the protein spatial structure stimulated by interfacial adsorption [20].

Therefore, this study aimed to gain a better understanding of the effects of different pH and salting on golden pompano myofibrillar protein emulsion gel by applying MDS on the structural conformation of myosin. The structural changes in myosin were observed under both confocal laser scanning microscopy (CLSM) and scanning electron microscopy (SEM). Finally, we analyzed the relationship between the observed structural changes with the changes in the emulsion gel rheology using computational simulation.

## 2. Results and Discussion

### 2.1. The Microscopic Morphology and Structure of Golden Pompano Myofibrillar Protein 

**Figure 1:**
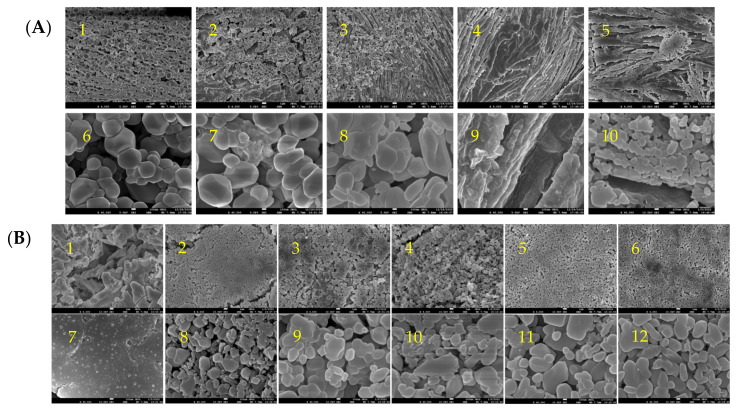
Microstructure of myofibrillar protein particles under scanning electron microscope: (**A**) microstructures at different pH (0.6 M NaCl; A1—pH 3.0, A2—pH 5.0, A3—pH 7.0, A4—pH 9.0, and A5—pH 11.0 at 4000× magnification; A6—pH 3.0, A7—pH 5.0, A8—pH 7.0, A9—pH 9.0, and A10—pH 11.0 at 40,000× magnification); (**B**) microstructures under different Na concentrations (pH = 7; B1—0 M, B2—0.20 M, B3—0.40 M, B4—0.60 M, B5—0.80 M, and B6—1.0 M at 4000× magnification; B7—0 M, B8—0.20 M, B9—0.40 M, B10—0.60 M, B 11—0.80 M, and B 12—1.0 M at 40,000× magnification).

Changes in pH can affect the charge distribution of amino acid side chains and thus influence the interactions between proteins. Therefore, the structure and properties of myofibrillar protein particles can be altered by adjusting the pH of the system. Figure 1A shows the microscopic aggregation of golden pompano myofibrillar protein under different pH treatments. At pH 3.0, the microscopic morphology of MP was observed (40,000× magnification) to be granular, which began to crosslink at a higher pH, forming aggregates at pH 5.0. At pH 7.0, the protein structure appeared to be fibrous, and the particles seemed to be ovalized when observed at high magnification. When the pH was 9.0, the protein fibrillization became apparent, while the granular particles became smaller. At pH 11.0, the granularity disappeared, and an abundant number of cross-linkages appeared across a large area of the protein. Our results are consistent with the findings by Sun et al. [18], which showed that myosin particles exhibit different shapes at different pH, with both hydrophobic interaction and electrostatic repulsion being cited as possible reasons for the phenomenon [21,22]. 

Salt can lower the isoelectric point of the myofibrillar protein, causing it to be net charged, which enhances solubility and thus improves its processibility [23]. Figure 1B shows the microscopic morphology of golden pompano myofibrillar protein under different salinities. Without NaCl (0.0 M), the MP was flaky and irregular. At 0.2 M NaCl, the proteins became granular and started to aggregate irregularly at 0.4 M NaCl. The aggregation continued at 0.6 M NaCl before stopping at 0.8 and 1.00 M NaCl, where the proteins formed into larger granules. The presence of salt also affects the amount of electrostatic charge around the protein. At low ionic strength, the shielding effect of free ions reduces the electrostatic repulsion between proteins [24], causing proteins to aggregate. With the increase in ionic strength, the interaction between salt ions and protein increases, facilitating the hydration of protein. At high ionic strength, the enhanced electrostatic repulsion between negatively charged myosin molecules resulted in the disappearance of aggregates.

### 2.2. Molecular Dynamics (MD) Simulations on pH and Salinity Effects

#### 2.2.1. MP Conformation Changes 

**Figure 2:**
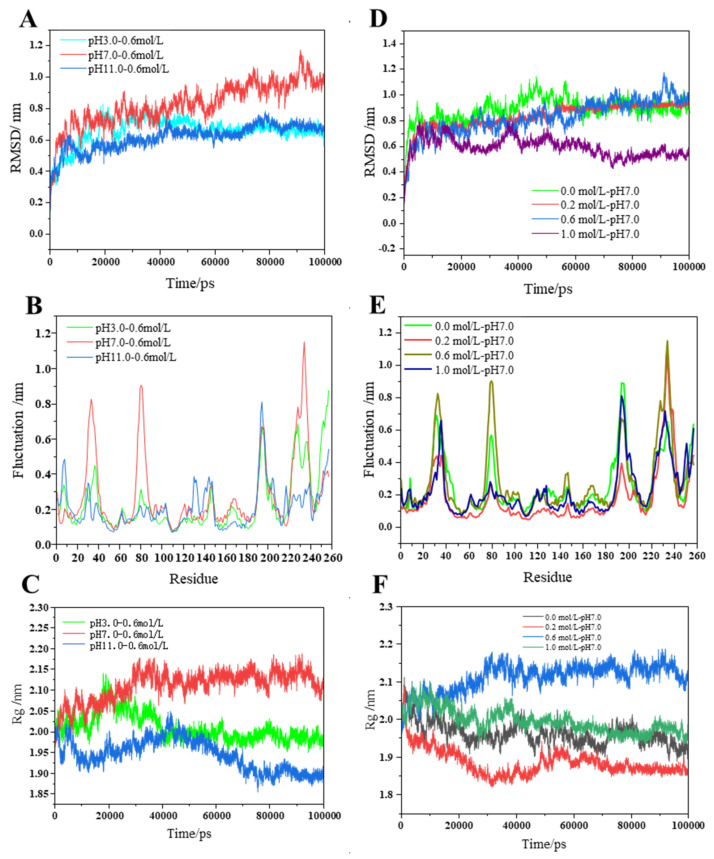
Structural behavior indicators over simulation time: (**A**) RMSD variation of apo structure heavy atoms; (**B**) RMSF of apo structure heavy atoms; (**C**) radius of gyration of apo structure; (**D**) RMSD variation of holo-structure heavy atoms; (**E**) RMSF of holo-structure heavy atoms; and (**F**) radius of gyration of holo-structure. RMSF x-axes denote i. the secondary structures, ii. the sub-domains, and iii. the disulfide bridges. The 4 °C systems are depicted with black lines; 25 °C systems are depicted with red lines; and 75 °C systems are depicted with green lines. (For interpretation of the references to color in this figure legend, the reader is referred to the web version of this article.)

The stability of the simulation system can be indicated by the root mean square deviation (RMSD) value, which measures the average deviation of the protein structure from the original conformation at a specific time. Therefore, changes in RMSD values would imply an alteration in protein conformation. As protein conformation plays a crucial role in emulsion gel stability [8], the influences of pH and salinity on MP conformation behavior were investigated by calculating its molecular dynamics trajectories of 100 ns under different pH (3, 7, and 11; NaCl 0.6 M) and NaCl (0.0, 0.2, 0.6, and 1.0 M; pH 7) concentrations. Our data (Figure 2A,D) show that the conformation of the protein has significantly deviated from its initial conformation under both types of treatment (pH and sodium), with equilibria mostly achieved after 80 ns. From the average value of RMSD after equilibrium, we found that the RMSD values were greater at pH 7.0, with an average value of 0.98 ± 0.06 nm after 80 ns. On the other hand, deviations at pH 3.0 and 11.0 were relatively small, suggesting that pH changes would impede alteration in protein conformation. Similarly, the average value of RMSD after 80 ns at 0.6 M NaCl was 0.98 ± 0.06 nm, which was slightly higher than 0.0 M (0.90 ± 0.04 nm) and 0.2 M (0.92 ± 0.01 nm) NaCl, indicating higher deviation from the original conformation of the protein. However, the average value of RMSD at a high salt concentration (NaCl 1.0 M) was significantly the lowest (0.54 ± 0.03 nm), suggesting that a system with excessive salt concentration would inhibit the shift in protein conformation. Taken together, the optimal condition of pH 7.0 and 0.6 M NaCl produced the greatest shift in MP protein structural conformation, thus allowing significant protein conformation changes. Hence, it can be said that both acid-base treatment and high salt concentration will inhibit the shift in MP protein conformation, which is not conducive to the conformational expansion of the MP protein structure (i.e., the formation of emulsion gel). 

#### 2.2.2. Changes in Protein Density

Given that emulsion gel formation is related to particle size [25], we proceeded to measure the protein density of myosin, which can be reflected in its radius of gyration (Rg) value. The smaller the radius of gyration, the denser the protein structure, and vice versa [26]. It can be seen from Figure 2C,F that myosin showed different trends in the extension of model time under different pH and NaCl concentrations. At pH 7.0, the overall radius of gyration increased (2.13 ± 0.02 nm), indicating the swelling of the protein, whereas the overall radius of gyration decreased at pH 3.0 (1.99 ± 0.01 nm) and pH 11.0 (1.90 ± 0.01 nm), signifying a more compact protein structure with the highest density at pH 11.0. At 0.6 M NaCl, the overall radius of gyration increased, and the protein swelled, while in other media of different ionic strength, the overall radius of gyration decreased, suggesting a denser protein structure.

#### 2.2.3. Fluctuation of Amino Acids Residues and Their Influence on Myosin Protein Size, Stability, and Structure

Following the results from the RMSD and Rg measurements, we determined that the conformation and density of myosin are influenced by changes in pH and NaCl concentration. As the degree of protein changes can be obtained based on the fluctuation in its amino acid residues, we then measured the root mean square fluctuation (RMSF) value, which denotes the positional change of each amino acid residue in a protein compared to the average conformation [27]. Figure 2B,E showed that the treatment with the most significant fluctuation of amino acid residues was pH 7.0 at 0.6 M NaCl, which is consistent with the results from the RMSD and Rg measurements. Some of the residues with larger RMSF under various pH include lysine (33 Lys and 194 Lys), alanine (80 Ala), valine (234 Val), tyrosine (257 Tyr), and others. In addition, residues with a larger RMSF in the NaCl system were serine (32 Ser), lysine (33 Lys and 194 Lys), glutamine (36 Gln), alanine (80 Ala), valine (234 Val), tyrosine (257 Tyr), and others (Figure S3). It may be that these residues are more flexible and thus were responsible for the observed protein swelling. 

**Figure 3:**
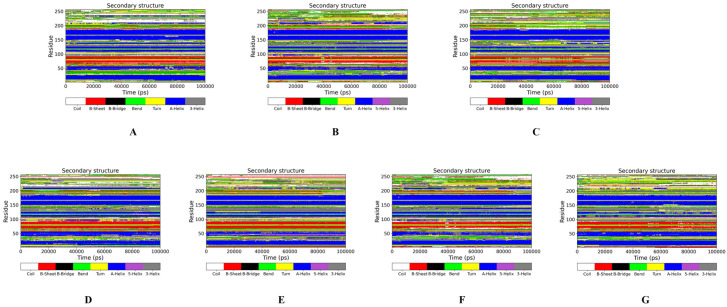

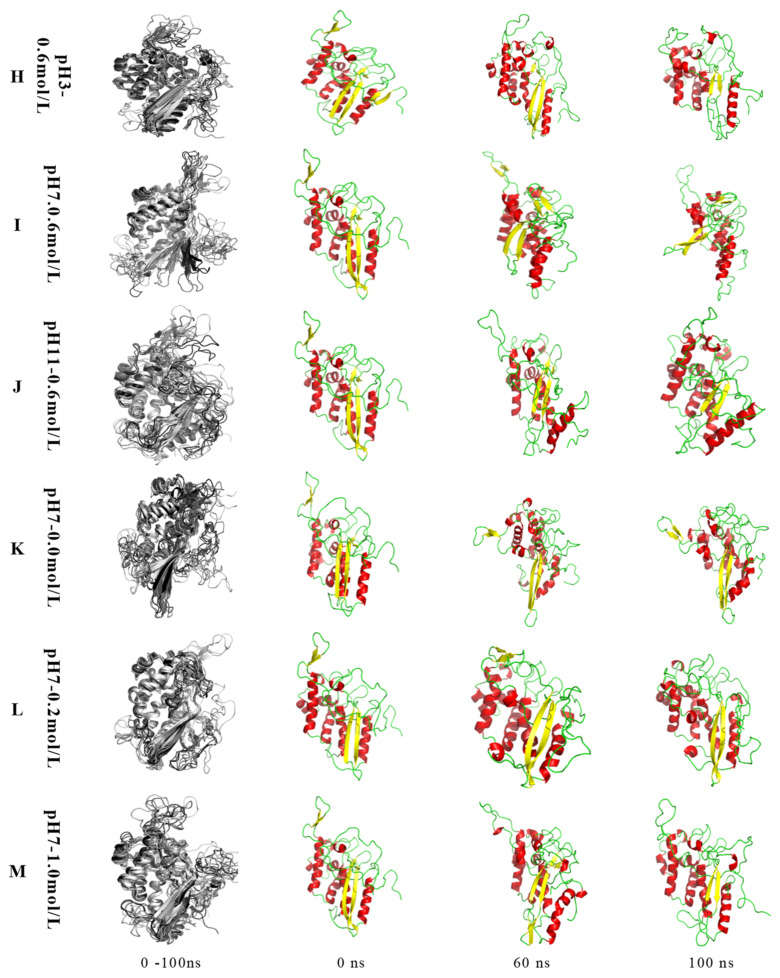
Secondary structure assignments by residue over simulation time for each system (**A**–**G**) and trajectory frames superposition at 0, 100, and 200 ns highlighting native secondary structures for each system (**H**–**M**).

To study in more detail how amino acid residue fluctuations affected myosin stability, the secondary structures for each system were assigned by residues and underwent simulations using DSSP and VMD algorithms. Trajectory frames at 0, 60, and 100 ns were sampled to monitor the behavior of all secondary structures in myosin (Figure 3). For myosin under pH manipulation, the superposition of myosin trajectories from 0 to 100 ns showed significant differences. The major residues that exhibited high fluctuation changes were β-sheets/turn at No. 71–77, No. 83–91, No. 187–191, and random secondary structures at No. 231–234. Among them, pH 3 caused the most fluctuations, which involved the deletion of β-sheet/turn at No. 2–4 and helical structures at No. 41–48 and 203–213, respectively. Fluctuations at pH 7 and 11 were also high, but no significant changes in secondary structures were observed. Notably, at pH 7, the fluctuating β-sheet/turn conformations exhibited a tendency to expand outward, which was consistent with the RMSF results.

The superimposed 0–100 ns trajectories for myosin under the manipulation of NaCl concentrations also showed differences in their structural conformation. However, the changes in the secondary structures at 0.0 and 0.2 M NaCl were not significant, even though there were some high atomic fluctuations. At 0.6 and 1.0 M NaCl, the β-sheet/turn of Nos. 71–77 and 83–91 showed high fluctuations but did not show any signs of expansion.

#### 2.2.4. Swelling of Myosin

**Figure 4:**
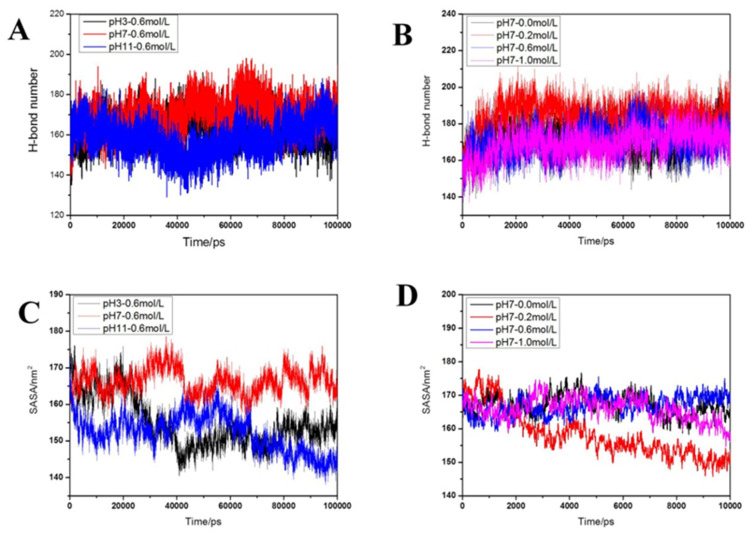
The H-bond number and SASA index changes under simulated system conditions: (**A**) H-bond number change under various pH; (**B**) H-bond number change under various NaCl concentrations; (**C**) SASA change under various pH; and (**D**) SASA change under various NaCl concentrations.

**Figure 5:**
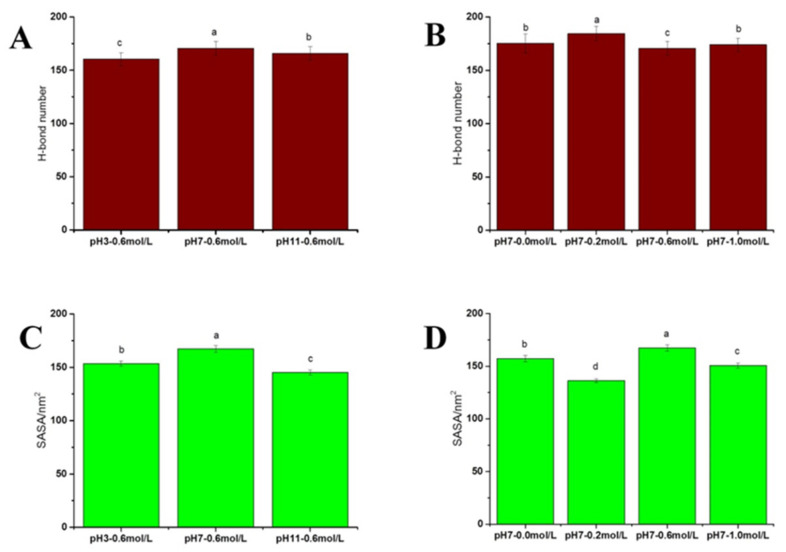
Changes in the H-bond number and SASA index at simulation time 80 ns–100 ns: (**A**) H-bond number under pH treatments; (**B**) H-bond number under NaCl treatments; (**C**) SASA index under pH treatments; and (**D**) SASA index under NaCl treatments.

Following the results of protein density, we further characterized the changes in myosin size induced by the treatments. Solvent-accessible surface area (SASA) can be considered a decisive factor in the folding and stability of proteins [28]. It can be seen from Figure 4C and Figure 5C that the SASA values were in the order of pH 7.0 > pH 3.0 > pH 11.0. Thus, the structural expansion of myosin was inhibited by the acid-base treatment, which could be explained by pH 7.0 being beneficial in increasing the contact surface area between the hydrophobic groups and water molecules, leading to the swelling of myosin. Figure 4D and Figure 5D show the SASA values at different salinities and were in the order of 0.6 M > 0.0 M > 1.0 M > 0.2 M NaCl. Notably, at 0.0 M NaCl, the SASA value of myosin was relatively high, which suggests the expansion of myosin structure under hydration. With the addition of sodium salt, the SASA value decreased due to electrostatic repulsion between proteins [29]. Under low sodium conditions (below 0.3 M), the sodium concentration was inversely proportional to the SASA value, which may be due to the fibrilization of the protein aggregates [24]. At 0.6 M NaCl, the myosin swelled, exposing the hydrophobic groups [30] and thus facilitating increased contact surface area between the hydrophobic group and water. In a high saline system, the salinization effect disrupts the charge distribution on the surface of the myosin structure, resulting in changes to the internal structure of the myosin molecule [31], leading to a lower SASA value.

#### 2.2.5. Distribution of Hydrogen Bonds

Hydrogen bonding is an important non-covalent force in maintaining protein stability. Changes in sodium salt concentration and pH alter the number and distribution of hydrogen bonds, inducing changes to the structure of myosin. Figure 4A,B show that during the simulation process, although the breaking and formation of hydrogen bonds in myosin resulted in continuous changes in their number, there were insignificant differences between the hydrogen bond fluctuations for each treatment. This suggests that changes in acidity and ion concentration had limited influence on the protein’s overall structure and conformation, leading to a relatively constant number of hydrogen bonds. Figure 5A,B show that under pH treatment, the order of the hydrogen bond number was pH 7.0 > pH 11.0 > pH 3.0. As fewer hydrogen bonds would inevitably reduce the stability of the protein, it can be deduced that the protein is most stable at pH 7.0. As for NaCl treatment, the average number of hydrogen bonds was ranked as 0.2 M > 0.0 M > 1.0 M > 0.6 M NaCl. The number of hydrogen bonds first increased and then decreased with the increase in salinity, which is consistent with the results reported by Dai et al. [32]. 

### 2.3. Influence of pH Value on Emulsion Gel System

Our previous results found that 2.5 wt.% MP (pH 7.0 and 0.6 M NaCl) with an oil ratio of 0.60–0.69 φ would form a desirable emulsion gel, with 0.68 φ producing emulsion gel with the best elastic modulus G″. In the CLSM images (Supplementary Materials, Figures S4 and S5), we can see that the proteins were wrapped around the oil phase in the emulsion gel, forming multiple W/O/W structures.

**Figure 6:**
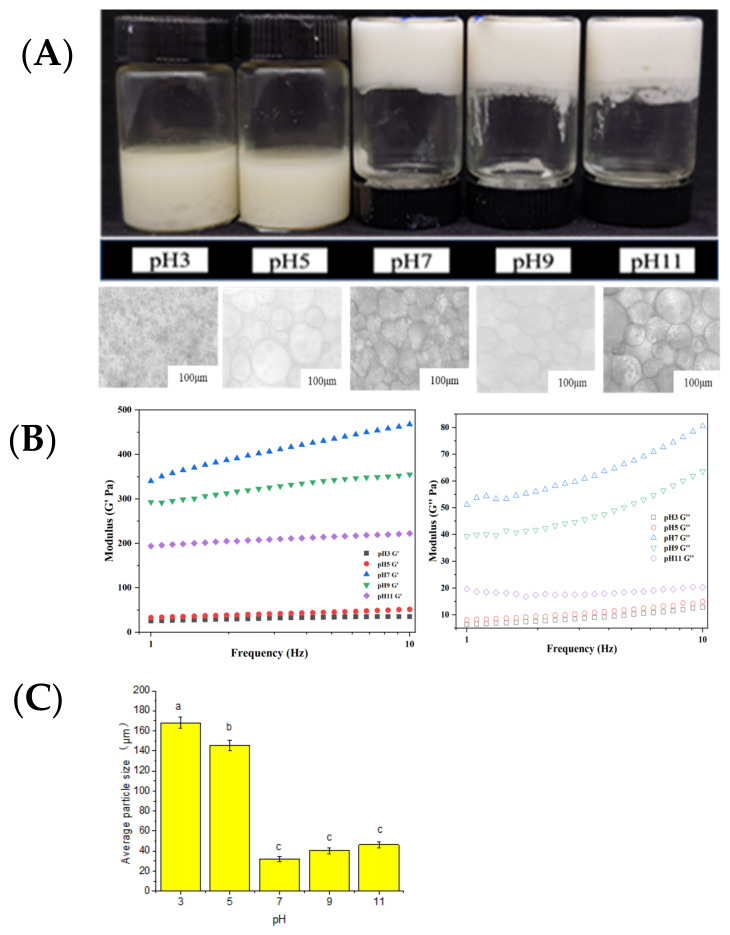
Characteristics of emulsion gel (protein concentration, c = 2.5 wt.% MP, and oil ratio ø = 0.68) under different pH. (**A**) Visual appearance (upper row) and optical microscope image (lower row) of emulsion gel under different pH (scale bar = 100 µm). (**B**) Effect of shear frequency (frequency 1–10 Hz and strain 0.5%) on elastic modulus (G’, solid) and loss modulus (G″, hollow) of emulsion gel under different pH (3, 5, 7, 9, and 11). (**C**) The average particle size of emulsion gel (d_3,2_); the letters denote significance as analyzed by Duncan’s multiple range test.

Figure 6A shows the morphology and optical microscopy of the myofibrillar protein emulsion gel (2.5 wt.% MP, φ = 0.68, and ionic strength = 0.6 M) at different pH values. When the pH was 7 (Figure 6B), the elastic modulus G″ of the emulsion gel was the best, followed by pH 9 and 11, while no emulsion gel was formed at pH 3 and 5. This indicates that the pH affects the spatial conformation of the myofibrillar protein [33], making it less available to bind oil and water. At pH 3.0, the microstructure of MP was observed to be granular. The molecular dynamics simulation results show that the protein structure was dense with a low SASA value (Figure 4C) and a reduced number of hydrogen bonds (Figure 4A). At the same time, the molecular dynamics trajectories showed significant reductions in β-sheet structures, which may result in poor protein emulsifying ability and hence the absence of emulsion gel formation. At pH 7, there was a large deviation in the protein conformation, as explained by the molecular dynamics simulation results. Furthermore, the trajectory diagram of molecular dynamics showed that the MP structure had expanded, resulting in the highest SASA value (Figure 4C) and the number of hydrogen bonds (Figure 4A), which are favorable for emulsification. Similarly, the elastic modulus (G″, solid) of the emulsion gel at pH 7 was also the largest. At pH 11, the microstructure of MP was in a cross-linked state with a low number of hydrogen bonds (Figure 4A) and SASA value (Figure 4C). Although the molecular dynamics simulation results show that the protein structure was dense, no significant loss of spatial structure and conformation was observed from the trajectory diagrams of the molecular dynamics. Therefore, we opined that the spatial structure and conformation state of the protein could affect the emulsification ability of the MP.

It can be seen from Figure 6C that the particle size of the emulsion is significantly larger in acidic systems (pH 3.0 and 5.0) when compared to neutral (pH 7.0) and alkaline (pH 9.0 and 11.0) systems. At pH 7.0, the particle size of the emulsion gel was the smallest (Figure 6C). In an acidic system, no emulsion gel was formed, which could be due to the presence of significantly larger particles with strong repulsive forces between them, preventing coalescence. As the pH increased, the decreasing particle size (possibly due to the alkaline effect on proteins) changed the balance of interacting forces, resulting in the spatial rearrangement between the myofibrillar protein particles that enable emulsion gel formation. The mutual repulsion between adjacent molecules at higher pHs then led to a slightly larger particle size [34,35].

### 2.4. Influence of Ionic Strength on Emulsion Gel

**Figure 7:**
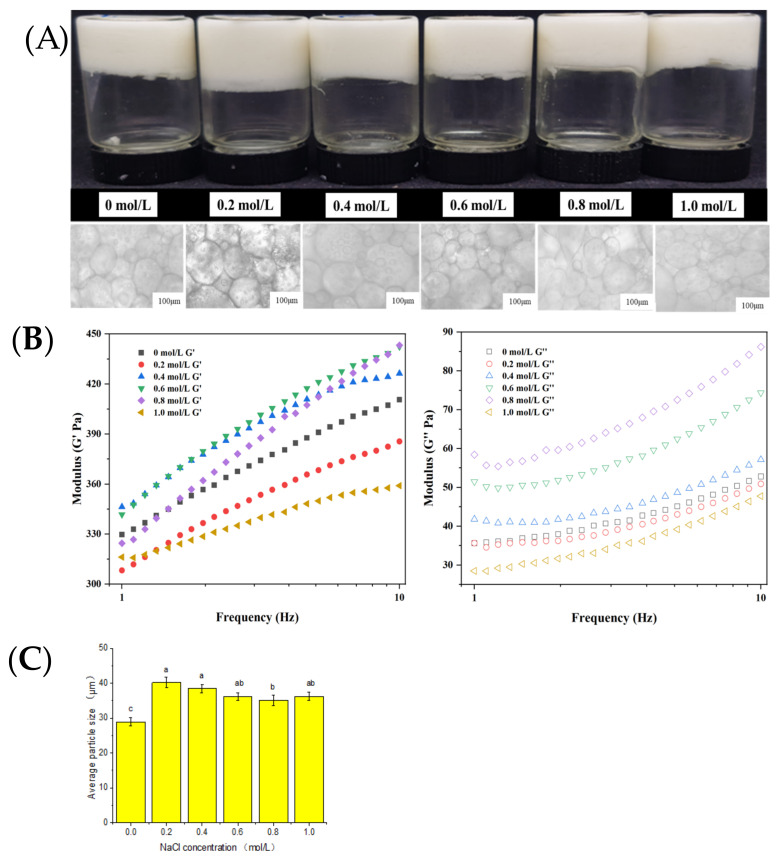
Characteristics of emulsion gel (protein concentration, c = 2.5 wt.%, and oil ratio ø = 0.68) under different NaCl concentrations. (**A**) Visual appearance (upper row) and optical microscope image (lower row) of emulsion gel under different NaCl concentrations (scale bar = 100 µ m). (**B**) The influence of shear frequency (frequency is 1 Hz, and strain is 0.5%) on the elastic modulus (G’, solid) and loss modulus (G″, hollow) of emulsion gel under different NaCl concentrations (0.2 mol/L, 0.4 mol/L, 0.6 mol/L, 0.8 mol/L, and 1.0 mol/L). (**C**) The average particle size of emulsion gel (d_3,2_); the letters denote significance as analyzed by Duncan’s multiple range test.

The morphologies and optical microscopies of emulsion gels (2.5 wt.% MP, pH 7, and φ = 0.68) under different ionic conditions are shown in Figure 7A. It can be seen that under the ionic strength of 0.0 to 1.0 M, the myofibrillar protein of golden pompano formed emulsion gel, denoting an insignificant effect of ionic strength on its emulsifying ability. At 0.6 M NaCl (Figure 7B), the emulsion gel showed the best elastic modulus G’, followed by samples at 0.8 M > 0.4 M > 0.0 M > 0.2 M > 1.0 M. The molecular dynamics trajectory diagram shows that the sodium salt concentration had a limited effect on the overall protein molecular structure and conformation, which was consistent with the observed microstructure of the MP. We also found that the particles largely remained small-sized despite occurrences of various cross-linking between them (Figure 1B). The mean protein RMSD and Rg results indicate that 0.6 M NaCl induced the largest conformational change, followed by 0.0 and 0.2, and finally, 1.0 M NaCl, all of which elucidated similar changes in the rheology of emulsion gel. The SASA value (Figure 4D) initially decreased before increasing, while the number of protein hydrogen bonds displayed an opposite trend with the increase in NaCl concentration.

In addition, our results (Figure 7C) suggest that NaCl concentration has a certain effect on the particle size of the emulsion gel. The particle size of the emulsion gel is the smallest at 0.0 M NaCl. At 0.2, 0.4, and 0.6 M NaCl, the particle size of the emulsion gel was relatively large but decreased slightly at 0.8 and 1.0 M. Therefore, even though sodium salt has little effect on the conformation of the MP protein molecule, the aggregation of protein molecules was nonetheless affected. At a 0.0 M Na concentration, the solubility of MP was low, displaying a flaky structure that necessitated the formation of smaller droplets to stabilize the oil phase. As the NaCl concentration increased, the dissolution of the MP became larger, enhancing the particulate morphology of the MP and thus increasing the particle size of droplets. The gel behavior under oscillation is included in Supplementary Materials (Figure S4B). Nevertheless, when the concentration of NaCl exceeds a certain threshold, a significant reduction of particle size in the emulsion gel may occur due to the transformation of myosin from the filament state to the monomeric state.

## 3. Conclusions

The impact of pH and salinity changes on the microstructure and conformation of myosin was investigated in this study. Our data have shown that the effects of pH and NaCl treatment on the spatial structure and conformation of myosin were more pronounced than their effects on the secondary structure, but pH has a greater effect on the structure of myosin compared to NaCl, including on its granularity, conformation, secondary structures, and spatial arrangement of amino acids. At pH 7 (0.6 M NaCl), myosin has an increased overall radius of rotation, large fluctuation of amino acid residues, large solvent-accessible surface area, and an expanded structural conformation, contributing to the stability of the emulsion gel. Our findings show that changes in pH value would affect the structural state of the protein emulsion, as demonstrated by the gel formation in neutral and alkaline environments but not under acidic conditions. Hence, pH plays a decisive role in determining the emulsification ability of MP. Salinity, on the other hand, does not affect the emulsification ability of MP, although it could affect the rheology, as evidenced by the high elastic modulus G′ observed at 0.6 M NaCl (pH 7). As such, we can conclude that the formation of myofibrillar protein emulsion gel is determined by the surrounding system and spatial conformation of the protein. At the same time, our study also supports the feasibility of molecular dynamics simulation as a valuable supplementary tool in analyzing structural changes in emulsion gel.

## 4. Materials and Methods

### 4.1. Materials

Commercial frozen golden pompano fillets (within 3 months from the date of harvest, 500 g, 15 cm × 8 cm) were supplied by Hainan Xiangtai Fishery Co., Ltd., Sanya, China. Deionized water (resistivity 18.2 MΩ·cm^−2^, 25 °C) was used for all experiments. All chemicals and reagents used were of GR grade purchased from Sinopharm Chemical Reagent Co., Ltd., Shanghai, China, including NaCl (≥99.8%), NaOH (≥99%), and hydrochloric acid (37%). 

### 4.2. Preparation of Golden Pompano Myofibrillar Protein 

The extraction of myofibrillar protein (MP) was performed according to the method described by Bakry et al. [36] with some modifications. Frozen fillets of golden pompano were thawed at 4 °C. After thawing, the white meat of the golden pompano was excised from both sides of the fish and crushed in an ice bath. The minced meat was sequentially centrifuged thrice (4000 rpm, 10 min, 4 °C) in a low-phosphate buffer solution (0.05 M NaCl, 3.38 mM NaH_2_PO_4_·2H_2_O, 15.50 mM Na_2_HPO_4_·12H_2_O, pH 7.5) and a high-phosphate buffer solution (0.6 M NaCl, 3.38 mM NaH_2_PO_4_·2H_2_O, 15.5 mM Na_2_HPO_4_·12H_2_O, pH 7.0). The mixture was then stored at 4 °C for 24 h before being centrifuged at 10,000 rpm for 10 min at 4 °C. The resulting supernatant was then collected in cold distilled water and rested at 4 °C for 120 min to allow for protein precipitation. The precipitated myofibrillar protein was then collected via centrifugation (10,000 rpm, 20 min, 4 °C), and stored in a refrigerator at 4 °C for use within 24 h.

### 4.3. pH and Salt Treatments on the Microstructure of Myofibrillar Protein

A total of 2.5 g of prepared pomfret MPs was dissolved in 100 mL of deionized water and was then subjected to different pH (3, 5, 7, 9, 11; ionic strength 0.6 M) and ionic conditions (0, 2, 4, 6, 8, 10 M; pH = 7) to obtain the myofibrillar protein solution. The protein solutions were then pre-frozen overnight at −60 °C before being freeze-dried for 12 h. The protein powder obtained was coated with gold particles and observed under SEM (JSM-7610F Plus, Hitachi, Tokyo, Japan).

### 4.4. Molecular Dynamics (MD)—The Effects of pH and NaCl on Myosin 

The golden pompano myosin sequence (UniProtKB: 1QWU5) was obtained from the UniProt database, while the templates of target structures were retrieved using the NCBI PSI-BLAST (Position-Specific Iterated BLAST) [37]. The best template (PDB ID: 4DB1) was obtained based on the calculated results for quality parameters, including species, the percentage of sequence coverage/probability, the E value, and the percentage of sequence identity. The 3D model of golden pompano myosin was constructed using Modeller 9.24 [38,39]. In brief, 100 modeling runs were performed under AllHModel, and the cycle was repeated 10 times until molpdf > 1 × 10^6^. Each model was first optimized using variable target function method (VTFM) with conjugate gradients (CG) and was then refined using molecular dynamics (MD) with simulated annealing (SA). Finally, the model with the best score was selected for further refinement using the locPREFMD algorithm (Figure S1 and Figure 2).

The optimal model was selected as the starting configuration for molecular dynamics simulation. Protonation discrimination of the protein was performed using Propka3 [40,41] according to the experimental conditions, and the corresponding ion concentration was added to the system. Molecular dynamics simulations were then performed for 100 ns using the GROMACS 19.6 package with Amber14sb force field for each explicit solvation system under different pH (3, 7, 11; 0.6 M Na salt) and ionic strength (0.0, 0.2, 0.6, 1.0 M Na salt; pH 7.0) [42]. Each system was placed in a cubic box of TIP3P water model with a minimum distance of 1.0 nm between any solute atom and the edge of the periodic box. Counter ions were added to neutralize the total charge to maintain a neutral system. Energy minimization (1000.0 kJ/mol/nm) was performed using the steepest descent. Subsequently, canonical ensemble (NVT, 1 ns) and isothermal-isovaric (NPT, 1 ns) were pre-balanced in the system successively [43]. All systems were simulated with 100 ns MD simulation, and all bonds containing hydrogen atoms were constrained using the default linear constraint (LINCS) solver algorithm [44]. The V-rescale thermostat [45] and Parrinello–Rahman barostat [29] were used to control the simulation temperature (298.15 K) and pressure (1.0 bar) with time constants of 0.1 and 2 ps, respectively. The Particle Mesh Ewald (PME) method [46] was employed to deal with long-range interactions, and a 10 Å cutoff was used for van der Waals interactions. The time step was 2 fs, and a snapshot was taken every 1.0 ps. After MDS, the GROMACS package (version 19.6) was used to analyze the trajectories. At the end of all simulations, the DSSP program [47] and VMD program [48] were used for secondary structure changes and visualization analysis, respectively.

Following the completion of the simulations, the periodic boundary conditions were removed, and the corresponding analysis commands of the Gromacs software were used to analyze the root mean square deviation (RMSD) and root mean square fluctuation (RMSF), the radius of gyration (Rg), secondary structure, solvent accessible surface area (SASA), and changes in the number of hydrogen bonds of the protein structure under different processing conditions. Among them, RMSD and radius of gyration were extracted from all 100 ns data, once every 0.1 ns, with a total of 1000 data points to analyze the structural changes of the protein during the whole simulation process, whereas the protein secondary structure, number of hydrogen bonds, solvent accessible surface area, and RMSF were only analyzed after protein stabilization, that is, data from 80 to 100 ns, with a total of 200 data points.

### 4.5. The Effects of pH and NaCl on Myofibrillar Protein Emulsion Gel System

Myofibrillar protein solutions were prepared under different pH (3, 5, 7, 9, 11; ionic strength 0.6 M, 2.5 wt.% MP) and ionic strength (0, 2, 4, 6, 8, 10 M; pH = 7, 2.5 wt.% MP). Corn oil (non-GMO, 100% purity) was added at φ = 0.68 and homogenized at 7000 rpm/min for 2 min under controlled temperature (<10 °C) to obtain the MP emulsion gel. The microstructure, rheology, and particle size of the emulsion gel were observed and measured.

### 4.6. Characterization of MP Emulsion Gel

#### 4.6.1. Microstructure of Emulsion Gel

The microstructure of the emulsion gel was determined using an optical microscope (B302, Optec, Chongqing, China). Samples (50 µL) of each emulsion were aspirated onto a glass slide and covered with a coverslip. Observation of the microstructure was undertaken at 40× magnification at room temperature. Furthermore, confocal laser scanning microscopy (CLSM) (FV3000, Olympus, Tokyo, Japan) was used to observe the oil–water interface of the emulsion gel according to the method described by Li et al. [7]. In brief, emulsion protein and lipid were, respectively, stained with 50 µL Nile blue (1 mg/mL dissolved in deionized water) and 50 µL Nile red (1 mg/mL dissolved in acetone) in the dark at a ratio of 25:1. The excitation wavelengths were 633 and 488 nm, respectively.

#### 4.6.2. Particle Size Measurements

The average particle size of the emulsion gel was determined using a laser diffraction particle size analyzer (LS13 320, Beckman Coulter, Lowell, MA, USA). The surface average droplet size (d_3,2_) of emulsion gel was taken as the average protein particle size.

#### 4.6.3. Dynamic Rheological Properties Analysis

The viscoelasticity of emulsion gel was measured using a parallel plate rheometer (MCR 92, Anton Paar, Graz, Austria; d = 49.953 mm) at 25 °C, with the gap between two plates set at 1.0 mm. The elastic modulus (G′) and loss modulus (G″) were determined under oscillation, in a range of frequencies (1 to 10 Hz) within the linear viscoelastic of 0.5% strain.

### 4.7. Statistical Analyses

All MD quantitative data were expressed as mean ± standard deviation. All images were composed using PyMOL and origin 8.5 software.

## Data Availability

The data presented in this study are available on request from the corresponding authors.

## Supplementary Materials

The following supplementary data can be downloaded at: https://www.mdpi.com/article/10.3390/gels9040270/s1, Figure S1: Evaluation of myosin structure in golden pompano as modeled by ab initio design using TrRosetta server; Figure S2: Schematic diagram of myosin structure optimization in golden pompano fish; Figure S3: Amino acid sequence of golden pompano myosin and the prediction of the secondary structure of golden pompano myosin using SignalP 5.0 and TPSPRED servers; Figure S4: Effect of oil ratio (φ) on emulsion gels; Figure S5: CLSM images of emulsion gel with 2.5% protein concentration, 0.68 oil ratio (φ), pH 7.0 and NaCl 0.6 mol/L.
