# Supplementary material for: Effects of pH and NaCl on the Spatial Structure and Conformation of Myofibrillar Proteins and the Emulsion Gel System—Insights from Computational Molecular Dynamics on Myosin of Golden Pompano
Source: Gels. 2023 Mar 24;9(4):270. doi: 10.3390/gels9040270 (PMC10137719; doi:10.3390/gels9040270)
Supplement: Supplementary file 1 [file gels-09-00270-s001.zip › gels-2282108-supplementary.pdf]

Supplementary Data

Effects of pH and NaCl on the Spatial Structure and Conformation of Myofibrillar Proteins and the Emulsion Gel System—Insights from Computational Molecular Dynamics on Myosin of Golden Pompano

Changfeng Xue [1,2,3], Zhisheng Pei [1,2,3], Pan Wen [2,3], Yaoxian Chin [1,2,3,4,*] and Yaqin Hu [1,2,3,*]

1 Hainan Provincial Academician Team Innovation Center, Hainan Tropical Ocean University, Sanya 572022, China
2 Marine Food Engineering Technology Research Center of Hainan Province, Hainan Tropical Ocean University, Sanya 572022, China
3 Collaborative Innovation Center of Marine Food Deep Processing, Hainan Tropical Ocean University, Sanya 572022, China
4 Yazhou Bay Innovation Institute, Hainan Tropical Ocean University, Sanya 572022, China
* Correspondence: chinyx1@163.com (Y.C.); yqhu@hntou.edu.cn (Y.H.)

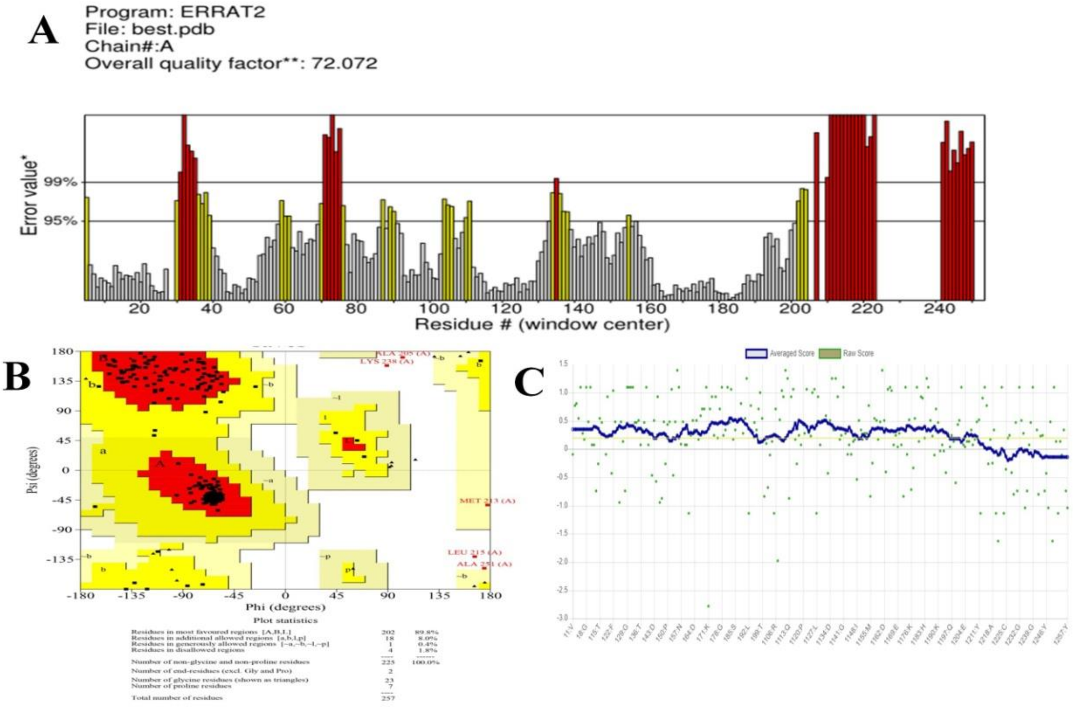

Figure S1. Evaluation of myosin structure in golden pomfret as modeled by ab initio design using TrRosetta server: (A) ERRAT analysis; (B) VERIFY3D analysis; (C) Ramachandran plots.

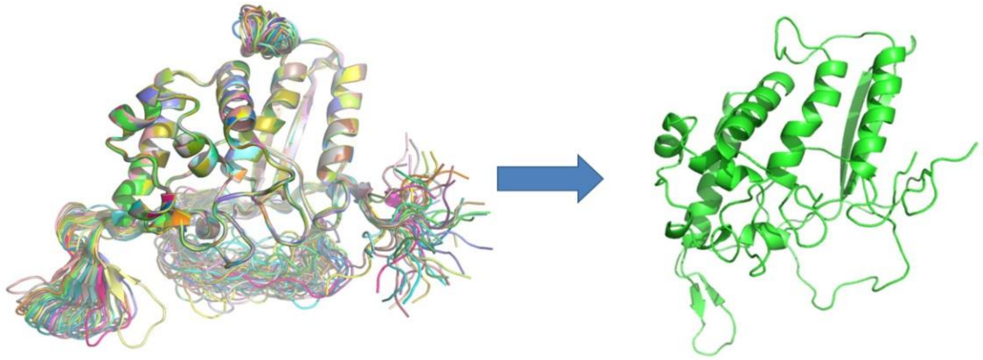

Figure S2. Schematic diagram of myosin structure optimization in golden pomfret fish.

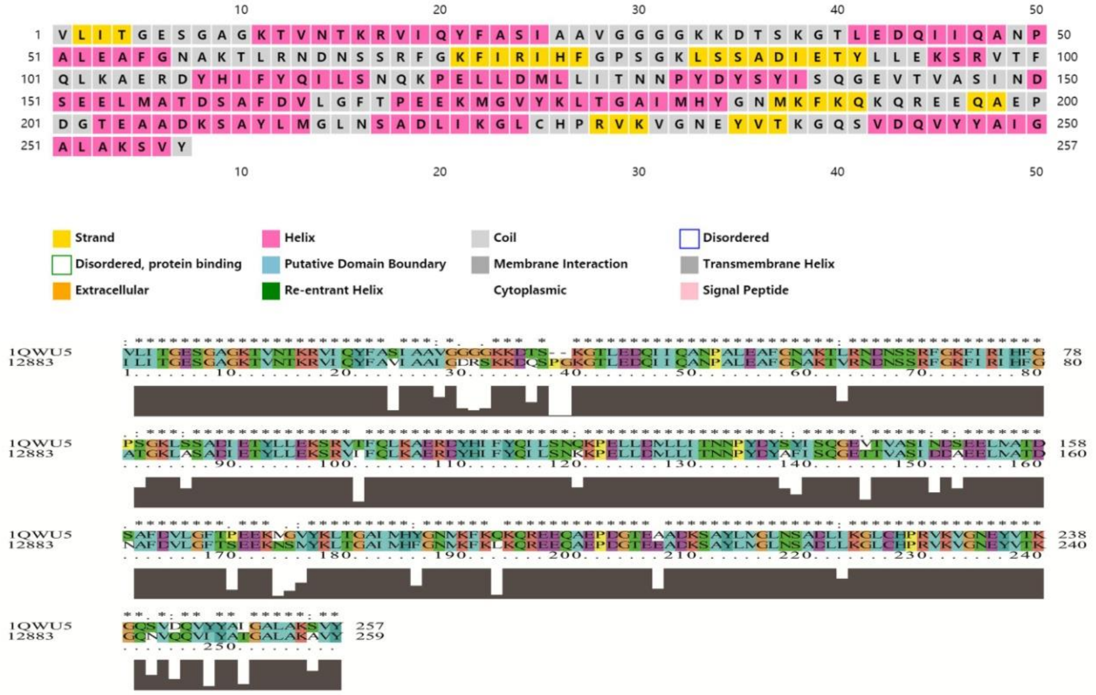

Figure S3. Amino acid sequence of golden pomfret myosin and the prediction of secondary structure of golden pomfret myosin using SignalP 5.0 and TPSPRED servers.

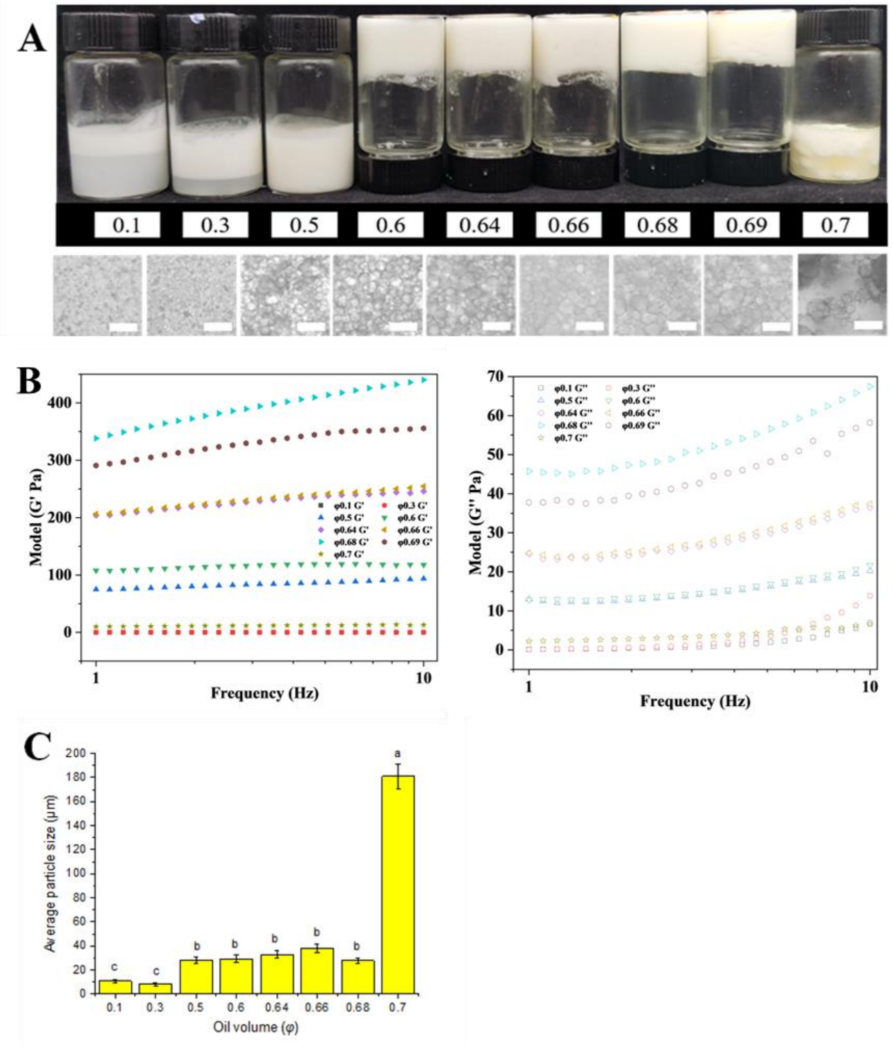

Figure S4. Effect of oil ratio (φ) on emulsion gels (c = 2.5 wt%, pH 7.0, NaCl 0.6 mol/L): (A) Visual appearances (top row) and light microscopy images (bottom row) of emulsion gels with different oil ratio (scale bar = 100 μm); (B) Effect of shear frequency (frequency 1-10 Hz, strain 0.5%) on the elastic modulus (G′, solid) and viscous modulus (G'', hollow) of emulsion gels with different oil ratios (0.10-0.70); (C) Average particle size of emulsion gel (d3, 2), the letters denote significance as analyzed by Duncan's Multiple Range test..

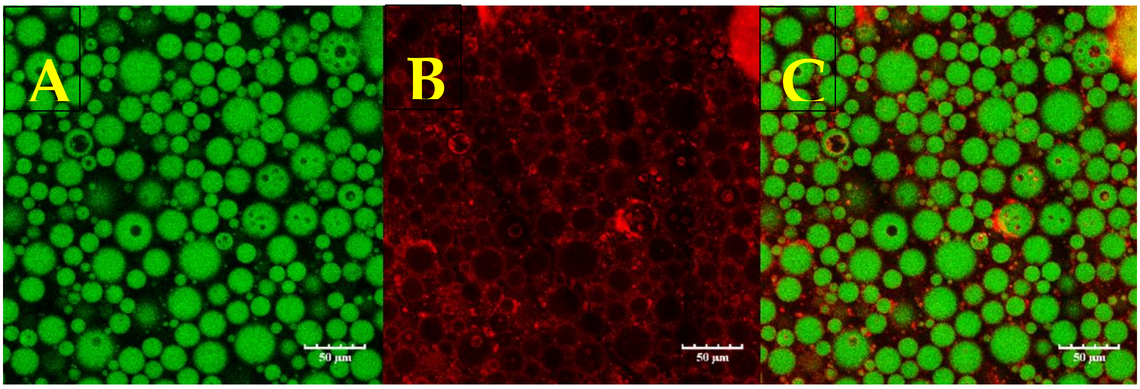

Figure S5. CLSM images of emulsion gel with 2.5% protein concentration, 0.68 oil ratio (φ), pH 7.0 and NaCl 0.6 mol/L. (A) Image with oil stain (Nile red); (B) Image with protein dye (Nile blue); (C) Image with double staining of oil and protein.
